# Chromium-Doped Magnetite Nanoparticles for Permanganate Ion Removal: Advances in Environmental Remediation

**DOI:** 10.1021/acsomega.5c12244

**Published:** 2026-05-18

**Authors:** Daniel de Lima Silva, Rubens Lucas de Freitas Filho, André Esteves Nogueira, Juliana da Fonseca Alves, Luiz Fernando Cappa de Oliveira, Guilherme Jorge Brigolini Silva, Rodrigo S. Corrêa, Adilson Candido da Silva, Ângela Leão Andrade

**Affiliations:** † Departamento de Química, Instituto de Ciências Exatas e Biológicas, 28115 Universidade Federal de Ouro Preto (UFOP), Ouro Preto, Minas Gerais CEP 35400-000, Brasil; ‡ Departamento de Química, Instituto de Ciências Exatas, 28114 Universidade Federal de Minas Gerais (UFMG), Belo Horizonte, Minas Gerais CEP 31270-901, Brazil; § Departamento de Química, Divisão de Ciências Fundamentais (IEF), Instituto Tecnológico de Aeronáutica (ITA), São José dos Campos, São Paulo CEP 12228-615, Brazil; ∥ Núcleo de Espectroscopia e Estrutura Molecular, Departamento de Química, 28113 Universidade Federal de Juiz de Fora (UFJF), Juiz de Fora, Minas Gerais CEP 36036-900, Brazil; ⊥ Departamento de Engenharia Civil, Escola de Minas, 28115 Universidade Federal de Ouro Preto (UFOP), Ouro Preto, Minas Gerais CEP 35400-000, Brasil

## Abstract

Iron mines with manganese contamination, as well as manganese mines, are found in various locations worldwide. During manganese ore extraction, permanganate ions (MnO_4_^–^) are released into water bodies, imparting an intense purple coloration that reduces light penetration, which can disrupt photosynthetic processes and negatively impact aquatic life. Additionally, their cytotoxic properties pose severe environmental and health risks. This study investigates the adsorption of MnO_4_^–^ onto Cr-doped magnetite nanoparticles (Cr-MNPs) as a potential remediation strategy for contaminated aqueous environments. Cr-MNPs and undoped magnetite nanoparticles were synthesized via the coprecipitation method and characterized using powder X-ray diffraction (XRD), revealing the presence of partially oxidized magnetite phases. X-ray photoelectron spectroscopy (XPS) analysis confirmed the high surface chemical purity of the materials, the presence of trivalent chromium at low concentrations, and the preservation of mixed Fe^2+^/Fe^3+^ surface species characteristic of magnetite, while revealing sample-dependent variations in surface oxygen species rather than a systematic increase in surface hydroxylation upon chromium incorporation. The adsorption process was evaluated by analyzing the effects of adsorbent dosage, contact time, and initial MnO_4_^–^ concentration, along with kinetic and isotherm modeling. Equilibrium was reached within 100 min, and experimental data were best described by the pseudo-second-order kinetic model and the Langmuir isotherm, indicating monolayer adsorption on a homogeneous surface. These findings demonstrate that Cr-MNPs are a promising adsorbent for MnO_4_^–^ removal, offering an efficient and sustainable solution for mitigating manganese contamination in aquatic environments, achieving a removal capacity of up to approximately 41 mg g^–1^.

## Graphical abstract

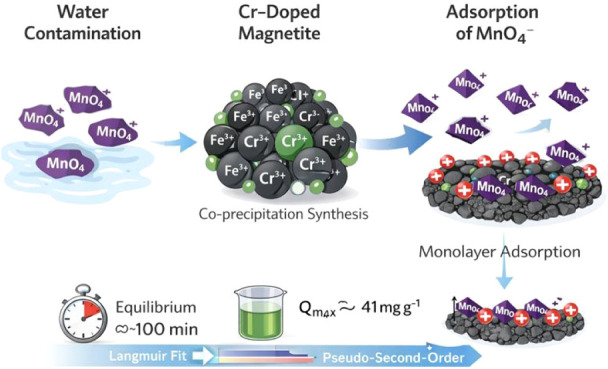

## 1 Introduction

The health status of a community, city, or state is evaluated using indicator-based methods, which serve as parameters for decision-making in public policy.^1^ Among these indicators are the infant mortality rate, basic sanitation coverage, and incidence of waterborne and airborne diseases.^2^ Water quality is fundamental to the assessment of basic sanitation coverage,^3^ significantly influencing improvements or deterioration in this parameter. The presence of toxic metals in effluents, unlike that of organic pollutants, which are largely biodegradable, is particularly concerning. Metallic ions are nonbiodegradable,^4^ and their excessive presence in water bodies threatens fauna and flora, with many carcinogenic properties. These metals can interact with macromolecules in biological membranes,^5^ accumulating in organisms and transforming normal concentrations to toxic ones.^6^ They can be stored in tissues, causing metabolic disturbances and ecological imbalances with long-term adverse effects even after emissions have ceased.

There are several alternatives for the removal of metallic ions, such as solvent extraction, reverse osmosis,^7^ ion exchange,^8^ precipitation,^9^ coprecipitation,^10^ and adsorption,^11^ which vary significantly in terms of the cost and technology complexity. Among these methods, adsorption stands out as an effective and low-cost technique. An interesting class of adsorption materials is based on nanoparticles, including magnetite, which stands out due to its unique, advantageous, and promising characteristics for use as an adsorbent. Its notable magnetic properties, combined with a significant specific surface area and low toxicity, enable the use of magnetic separation processes for removing saturated adsorbent material. In addition to their magnetic properties, iron-based materials can be easily separated because of their high density, which promotes sedimentation and decantation in aqueous media.

Several recent review papers have provided comprehensive overviews of advances in magnetic iron oxide-based nanomaterials for water purification. These studies discuss how factors such as nanoscale architecture, surface chemistry, and synthesis routes influence adsorption efficiency and practical applicability.^12–14^ They also outline the fundamental adsorption mechanisms of iron oxides and underscore their advantages as effective, magnetically recoverable sorbents for the removal of inorganic contaminants. Collectively, these insights provide important guidance for the design of more robust and tunable magnetic adsorbents for environmental remediation.

To expand the technological applications of magnetic nanoparticles and incorporate atoms with compositions distinct from those of magnetite into its crystalline structure, a doping approach was selected. This choice is based on well-established methodologies reported in the literature that provide a robust foundation for the controlled development and manipulation of these materials.

Several studies have reported the adsorption of various metals using magnetite has gained prominence, including Pb­(II), Zn­(II), and Cd­(II);^15^ As and Cu;^16^ Cr­(VI) and Cu­(II),^17^ as well as Cd­(II) and Pb­(II).^18^ In this study, Cr­(III) was selected for incorporation into the magnetite crystalline structure. The aim of this work is to evaluate the performance of Cr-doped magnetite as an adsorbent by analyzing adsorption isotherms and assessing its efficiency in removing permanganate ions (MnO_4_^–^) from synthetic solutions that simulate concentrations found in water impacted by manganese-rich mining activities.

Therefore, MnO_4_^–^ was chosen in this study due to the following factors: (i) Manganese deposits are found in various locations within the municipality of Ouro Preto;^19^ and (ii) during ore extraction, MnO_4_^–^ ions may leach into the region’s water bodies. As a result, contaminated water can adversely affect the environment and human health due to its carcinogenic and cytotoxic properties.^20^ Given its deleterious effects, this pollutant is included in the priority pollutant list of the Environmental Protection Agency.^21^ Thus, simultaneous detection and remediation of this anion from aqueous streams are of great importance for environmental protection, yet they remain challenging.

## 2 Experimental Section

### 2.1 Materials

All chemicals used, such as ferric chloride hexahydrate—FeCl_3_·6H_2_O (Riedel-de Haën, France), chromium­(III) chloride hexahydrate—CrCl_3_·6H_2_O (Riedel-de Haën, France), sodium sulfite—Na_2_SO_3_ (Sigma-Aldrich, Japan), ammonium hydroxide—NH_4_OH (Fluka, Germany), and hydrochloric acid—HCl (Sigma-Aldrich), were of analytical grade and used as received.

### 2.2 Synthesis of Adsorbates

#### 2.2.1 Rapid Method for Synthesizing Magnetite Nanoparticles (MR) and Cr-Doped Magnetite Nanoparticles (MR1 and MR2)

Essentially, the method described by Andrade et al.^22,23^ was employed. Briefly, a 1 mol L^–1^ solution of sodium sulfite (Na_2_SO_3_) and a 2 mol L^–1^ solution of ferric chloride (FeCl_3_) were mixed and stirred until the solution color changed from dark yellow to light yellow. Then, 400 mL of diluted ammonium hydroxide (NH_4_OH) solution was added, resulting in the formation of a black precipitate. The suspension was centrifuged at 3500 rpm for 3 min. The supernatant was discarded, and the black precipitate was washed in distilled water and centrifuged. This procedure was repeated three times, and the resulting precipitate was labeled as “MR.” For the MR1 and MR2 samples, the preparation method was similar to that of the MR sample, but about 0.3 and 1.5 g of CrCl_3_·6H_2_O, respectively, were added to the solution of FeCl_3_ before mixing with Na_2_SO_3_.

#### 2.2.2 Synthesis of Aged Magnetite Nanoparticles (ME) and Cr-Doped Magnetite Nanoparticles (ME1 and ME2)

In this case, magnetite nanoparticles were synthesized in the same manner as described above, but alkalization was achieved by slowly adding a 0.5 mol L^–1^ NH_4_OH solution until a pH of 8.2 was reached. After pH adjustment, this mixture was transferred to a Schlenk flask reactor and purged with argon for 5 min. This flask was connected to another Schlenk flask containing a concentrated NH_4_OH solution. The system was left undisturbed at 62.5 °C for 8 days. The suspension was centrifuged at 3500 rpm for 10 min. The supernatant was discarded, and the black precipitate was washed with distilled water and centrifuged. This procedure was repeated three times, and the resulting precipitate was labeled as “ME” For the ME1 and ME2 samples, the preparation method was similar to that of the ME sample, but about 0.3 and 1.5 g of CrCl_3_·6H_2_O were added to the solution of FeCl_3_ before mixing with Na_2_SO_3_.

### 2.3 Characterization

The morphology of the samples MR and ME was determined by transmission electron microscopy using a thermionic-source (LaB6) transmission electron microscope Tecnai G2-20 SuperTwin (FEI), operated at 200 kV. The functional groups and chemical compounds present in the samples were identified by ATR–FTIR spectroscopy using a Mattson Galaxy S-7000 FT-IR spectrometer, with 32 scans and a spectral resolution of 4 cm^–1^. The atomic absorption analysis of all the Cr-doped samples was obtained in a spectrometer, Varian AA-175 Series. Powder X-ray diffraction analysis was performed using a Bruker D2 Phaser Second Generation diffractometer (Bruker Company, USA) with CuKα radiation (λ = 1.54184 Å), operating at 30 kV and 10 mA, scanning range from 7 to 70° with a step size of 0.02°, 1 s per step, for identification of crystalline phases. Rietveld refinement was conducted using the FULLPROF SUITE 2019 program, available at https://www.ill.eu/sites/fullprof/, with CIF files extracted from the Inorganic Crystal Structure Database (ICSD) and Jade+ software (Materials Data, Inc.) for phase identification. XRD measurements and Rietveld refinements were performed both before and after the adsorption experiments with KMnO_4_ in order to verify the structural stability of the iron oxide phase and to ensure that no phase transformation occurred during the adsorption process.

The elemental surface composition and corresponding oxidation states were analyzed by X-ray photoelectron spectroscopy using a Thermo Scientific K-Alpha instrument equipped with a monochromatic Al Kα X-ray source (hν = 1486.6 eV). Binding energies were calibrated against the C 1s peak of adventitious carbon at 284.8 eV. Data fitting and quantitative analysis were carried out with CasaXPS software (version 2.3).

Nitrogen adsorption/desorption (BET) analyses were performed on degassed samples at 70 °C for 4 h under vacuum using an Autosorb iQ at −196 °C, with a relative pressure range of 0.005 to 1.0. Approximately 300 mg of material was used, and the analysis time was 4 h. Surface area was estimated using the BET method, and pore size distribution was determined using the BJH method, employing ASiQwin 5.21 software. Micropore volume and area were determined using the t-plot method. The specific areas, measured using the BET method, were based on the adsorption of a monolayer of gas.^24^

Raman spectroscopy was performed using a dispersive spectrometer, Bruker, model SENTERRA. The three available excitation lines were used, 785, 633, and 532 nm, at 5 mW (785 nm) and 2 mW (633 and 532 nm) of laser power, using a 50× magnification objective and 3 cm^–1^ of spectral resolution. Due to sample sensibility, it was accumulated 15 spectra with 5 s of exposure time.

### 2.4 Adsorption Procedure

#### 2.4.1 Adsorption Kinetics

Kinetic assays were carried out by shaking 0.01 g of each adsorbent material (ME, ME1, ME2, MR, MR1, and MR2) with 50 mL of a 50 mg L^–1^ solution containing MnO_4_^–^ at different times, ranging from 15 to 1440 min. Later, the reaction mixtures were centrifuged for 10 min at 3500 rpm, and the remaining concentration was monitored through UV/vis spectroscopy at 526 nm.

In order to describe the kinetics of MnO_4_^–^ adsorption onto Cr-doped and undoped magnetite nanoparticles, characteristic constants were determined using the pseudo-first order equation of Lagergren^25^ and the pseudo-second order equation of Ho and McKay.^26^ The used kinetic model expressions are also presented in Table 1, where *c*_*e*_ is the equilibrium concentration of MnO_4_^–^ in the liquid phase (mg L^–1^), *q*_*e*_ is the MnO_4_^–^ adsorbed at equilibrium state (mg g^–1^), *q*_*max*_ corresponds to the maximum adsorption capacity of MnO_4_^–^ on samples (mg g^–1^), and *K*_*L*_ (min^–1^) and *K*_*F*_ (g mg^–1^ min^–1^) are the kinetic rate constants of the pseudo-first order and pseudo-second order, respectively.

**Table 1:** Mathematical Equations of the Used Isotherm and Kinetic Adsorption Models

Model	Equation	Parameter and dimension
Isotherm models		
Langmuir	$q_{e}=\frac{K_{L}q_{max}C_{e}}{1+K_{L}C_{e}}$	*q_max_* (mg g^–1^)
		*K_L_* (L mg^–1^)
Freundlich	$q_{e}=K_{F}C_{e}^{1/n}$	*K_F_* (mg g^–1^)
		*n* = model exponent
Kinetic models		
*Pseudo*-first order (Lagergern)	*q_t_* = *q_e_* (1 – exp(−*k_f_t*))	*k_f_* (min^–1^)
		*q_e_*,*q_t_* (mg g^–1^)
*Pseudo*-second order (*H*_0_)	$q_{t}=\frac{K_{s}q_{e}^{2}t}{1+q_{e}K_{s}t}$	*K_s_* (min^–1^)
		*t*: time (min)

The amount of MnO_4_^–^ adsorbed at equilibrium *q*_*e*_ (mg g^–1^) was obtained following Equation 1.

1 $$q_{e}=\frac{\left(C_{0}-C_{e}\right)V}{m}$$

where *C*_*e*_ (mg L^–1^) is the equilibrium concentration of MnO_4_^–^ solution, *C*_0_ (mg L^–1^) is the initial concentration of MnO_4_^–^, *V* (L) is the volume of the solution, and *m* (g) is the mass of the adsorbent.

Further, at time *t*, the quantity of adsorbed MnO_4_^–^ *q*_*t*_ (mg g^–1^) was calculated using Equation 2:

2 $$q_{t}=\frac{\left(C_{0}-C_{t}\right)V}{m}$$

where *C*_0_ (mg L^–1^) is the initial concentration of MnO_4_^–^ solutions, and *C_t_* (mg L^–1^) is the MnO_4_^–^ concentration at time *t*.

The values of linear coefficient regression (*R* ^2^) and $\chi_{red}^{2}$ are used to predict the most suited kinetic model for the adsorption process.

#### 2.4.2 Adsorption Isotherms

The adsorption isotherms for MnO_4_^–^ on the samples ME, ME1, ME2, MR, MR1, and MR2 (adsorbents) were carried out in batch adsorption experiments at room temperature. Ten mL of potassium permanganate (KMnO_4_) solution with different concentrations (from 5 to 150 mg L^–1^) were placed in contact with 10.00 mg of adsorbent in amber flasks under constant stirring at 180 rpm. The pH of the suspension was adjusted by adding either 0.1 mol L^–1^ NaOH or 0.1 mol L^–1^ HNO_3_. Adsorption tests were carried out at pH values of 5.6. The suspension was allowed to react for 3 h to reach equilibrium, and then the mixtures were centrifuged for 10 min at 3500 rpm, and the remaining concentration was monitored through UV/vis spectroscopy at 526 nm.

To ensure the accuracy, reliability, and reproducibility of the collected data, all batch tests were performed in triplicate, and average values only were reported. Blank tests were run in parallel on MnO_4_^–^ solutions without the addition of sorbent, showing that the experimental procedure does not lead to any reduction of MnO_4_^–^ and pH variation unrelated to sorbent effects.

Two isotherm models—Langmuir^27^ and Freundlich^28^ were employed to describe the adsorption equilibrium of MnO_4_^–^ ions onto Cr-doped and undoped magnetite nanoparticles. The mathematical formulations of these models are presented in Table 1, where *q* represents the amount adsorbed, and *C* denotes the equilibrium concentration of Cr­(III). According to the Langmuir model, *q*_*max*_ corresponds to the completion of a monolayer, while *K*_*L*_ is a constant related to adsorption energy. This parameter reflects the enthalpy of adsorption and acts as an indicator of the binding energy during surface adsorption. In the Freundlich model, *K*_*F*_ is a parameter representing the adsorption capacity. The term 1/*n* characterizes the adsorption intensity, indicating whether the adsorption is favorable (1/*n* < 1) or not (1/*n* > 1).

The intraparticle diffusion model was also studied.^29,30^ The intraparticle diffusion (IPD) kinetic model (Equation 3) represents in detail that there may be one or more steps involved in the adsorption process. First, the adsorbate molecules approach the external surface of the solid (external diffusion) and then undergo diffusion through the thin film of solvent molecules surrounding the adsorbent particles. After, the adsorbate molecules undergo diffusion to the internal pores and the surface of the solid (intraparticle diffusion).

3 $$Q_{t}=k_{it}^{0.5}+C$$

where *k_it_* (mg g^–1^ min^–1/2^) is the intraparticle diffusion rate constant and *C* is the intercept, which may be related to the thickness of the boundary layer.

To evaluate the parameters of isotherm and kinetic models, the nonlinear method is generally more effective than the linear one.^31,32^ Unlike the linear approach, which presumes that experimental data points are evenly distributed along a straight line and follow a Gaussian error distribution—an assumption rarely valid for equilibrium isotherm models—the nonlinear method relies directly on raw experimental data. This avoids the distortion of error distribution caused by transforming data into a linear format, an issue inherent to the linear method.^32^ In this study, MnO_4_^–^ adsorption data were analyzed using kinetic and isotherm models through ORIGIN 2018, employing a nonlinear approach.

## 3 Results and Discussion

### 3.1 Characterization of Cr-Undoped Magnetic Nanoparticles

Figure 1 compares the FTIR spectra of MR and ME samples. The spectra show the characteristic O–H stretching vibration of surface hydroxyl groups and adsorbed water (3200–3600 cm^–1^), the H–O–H bending mode at approximately 1600 cm^–1^, and a second absorption band, between 900 and 1000 cm^–1^, and the Fe–O stretching vibrations associated with the inverse spinel structure of Fe_3_O_4_ (*ca.* 580 and 450 cm^–1^).^33,34^ In addition, weak absorption bands observed around 1380 cm^–1^ and 880 cm^–1^ are attributed to the asymmetric stretching (ν_3_) and out-of-plane bending (ν_2_) modes of carbonate (CO_3_^2–^) species, respectively, arising from the adsorption of atmospheric CO_2_ on the surface of the nanoparticles, a phenomenon commonly reported for iron oxide materials synthesized under alkaline conditions.^35^ Although both MR and ME samples exhibit the characteristic vibrational features of magnetite, noticeable differences are observed in their FTIR spectra, particularly in the regions associated with surface-related vibrations. These differences provide important insights into the relative particle size, surface chemistry, and degree of structural order of the nanoparticles.^33^

**Figure 1:**
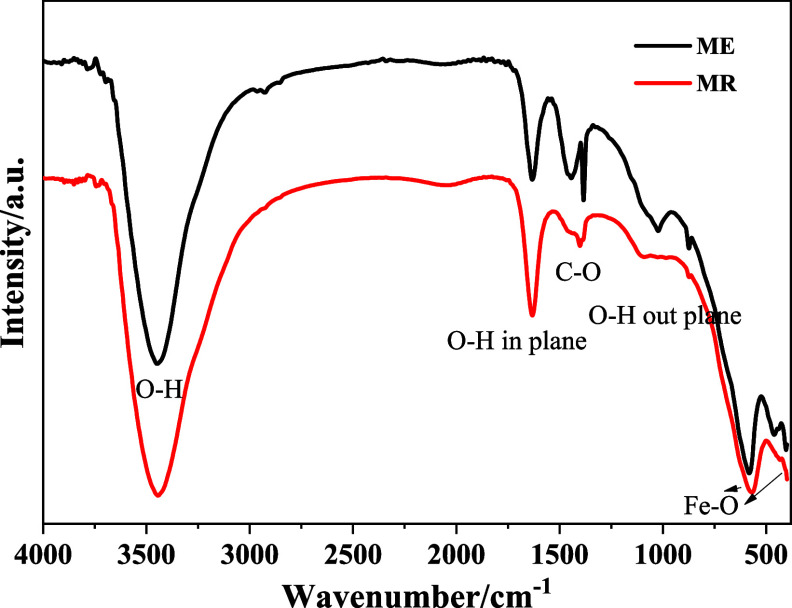
FTIR spectra of MR and ME samples.

A broad and intense absorption band in the 3200–3600 cm^–1^ region, assigned to O–H stretching vibrations of surface hydroxyl groups and adsorbed water molecules, is significantly more pronounced in the MR sample. In addition, the bending vibration of molecular water, observed around 1620–1650 cm^–1^, also exhibits higher intensity for MR. The enhanced intensity and broadening of these bands indicate a higher density of surface hydroxyl groups and physically adsorbed water, which is commonly associated with a larger specific surface area. This behavior strongly suggests that the MR sample consists of smaller nanoparticles compared with ME.^36^

In the low-wavenumber region (400–700 cm^–1^), both samples display the characteristic Fe–O stretching vibrations of the inverse spinel structure of magnetite, confirming the preservation of the crystalline phase in both materials. However, the Fe–O band in MR is noticeably broader and less well-defined than that observed for ME. Such band broadening reflects increased local structural disorder and a greater contribution from surface atoms, which is consistent with reduced particle size and a higher surface-to-volume ratio.^33^ In contrast, the sharper and better-resolved Fe–O vibration in the ME sample indicates a higher degree of crystallinity and a more ordered local environment, consistent with larger particle size or improved crystal growth.^33^

Importantly, no additional absorption bands associated with secondary iron oxide phases or surface contaminants were detected in either spectrum, indicating that the observed spectral differences arise predominantly from size-related surface effects rather than from changes in chemical composition or crystal structure. The FTIR results suggest that while both MR and ME are composed of magnetite nanoparticles, MR exhibits a higher degree of surface hydration and local structural disorder, characteristic of smaller particles, whereas ME presents a more ordered structure with a reduced surface contribution, consistent with larger nanoparticles.

The nanocrystalline structures of the synthesized Cr-undoped magnetic nanoparticles (MR and ME samples) were identified by powder XRD analysis. According to the results, it is observed that the two spectra are similar. The Rietveld refinement of the XRD patterns was performed according to the Fd3m space group pseudo-Voigt function, and the resulting Rietveld profiles are displayed in Figure 2. The quality of the Rietveld refinement was evaluated from the profile factor (*R*_p_), weighted profile factor (*R*_exp_), goodness of fit indicator (*S*), Bragg factor (*R*_B_), and crystallographic *R*_F_ factor parameters (Table 2). These results indicate the good quality of the structural refinement.

**Figure 2:**
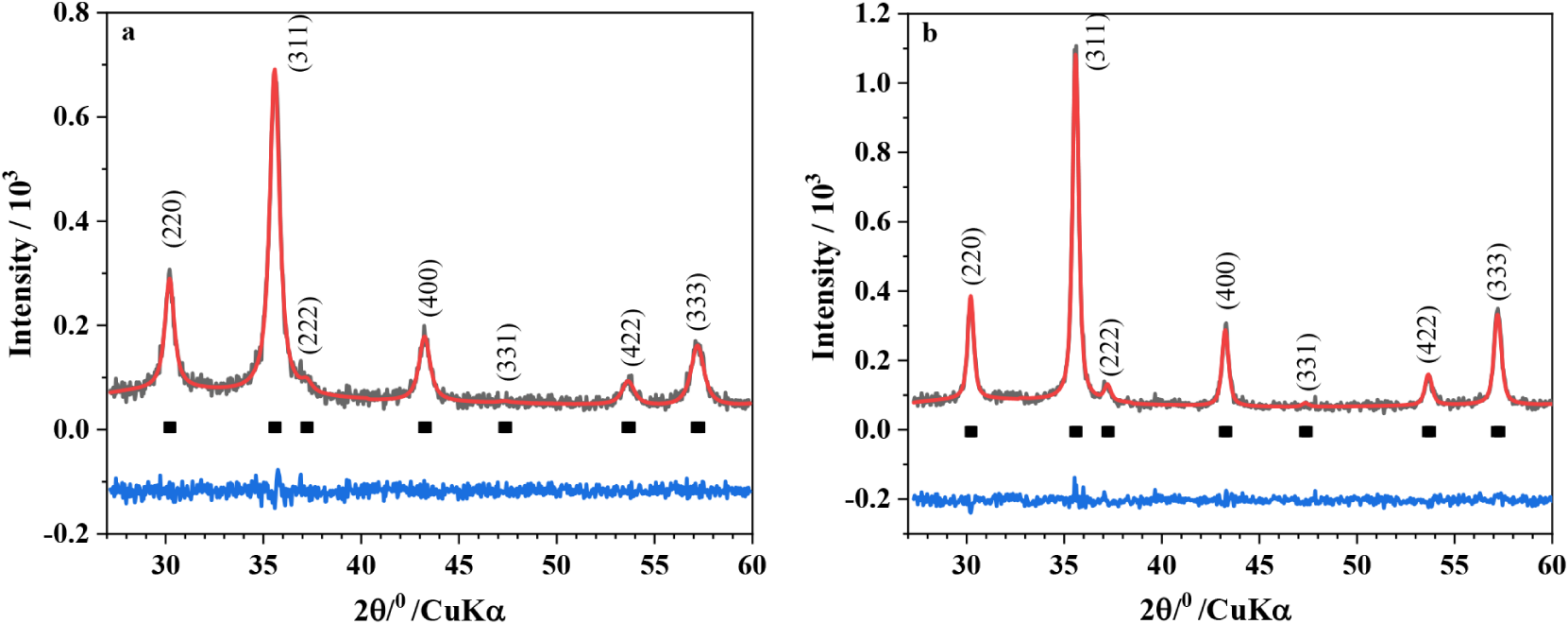
XRD patterns of the samples Cr-undoped: (a) MR and (b) ME.

**Table 2:** Structural Parameters and Rietveld Agreement Factors for Samples Cr-Undoped, MR and ME

	Sample	
Parameter	MR	ME
A	8.369 ± 0.003	8.367 ± 0.001
*R*_p_	7.71	6.25
*R*_exp_	10.3	8.22
*S*	0.99	0.84
*R*_B_	2.94	2.71
*R*_F_	2.47	2.53

The calculated unit cell dimensions of *a*_0_ ≈ 8.39 Å are typical of stoichiometric bulk magnetite, while the cubic maghemite has *a*_0_ ≈ 8.34 Å.^33,37^ On the other hand, magnetite nanoparticles prepared by basic precipitation from Fe^2+^/Fe^3+^ solutions commonly exhibit smaller lattice constants of 8.38 Å ≥ *a*_0_ ≥ 8.34 Å.^37^ This is expected given that a small particle tends to decrease the network parameters, as they oxidize more easily; the degree of oxidation is increased with decreasing particle size.^37^ In this work, we observed the occurrence of a single crystallographic phase of partially oxidized magnetite for both MR and ME samples.

Figure 3 presents the MR and ME Raman spectral results. Both spectra were acquired under identical measurement conditions for the two samples. The poor quality of the 785 nm Raman spectra in both cases is expected because of the sensitivity of Fe-based materials to infrared excitation sources. However, the remaining data were still analyzed. The spectra reveal a stronger Raman scattering signal for the sample ME compared to MR. This difference can be attributed to the greater resistance of the ME sample to laser power, which may be linked to the presence of larger particles. When comparing the two methods, the longer processing time to obtain the ME material can contribute to the formation of larger particles, as confirmed by the Raman spectral results.

**Figure 3:**
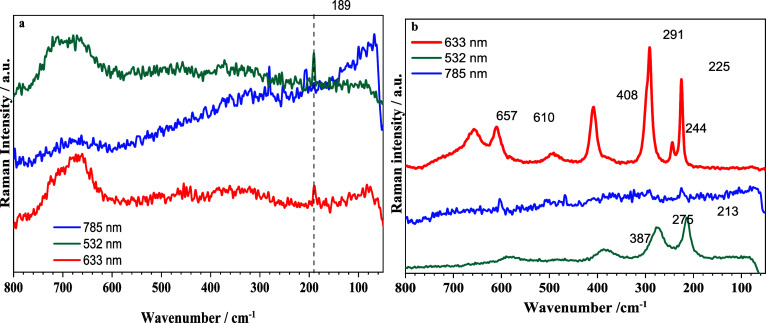
Raman spectra of the samples Cr-undoped: (a) MR and (b) ME, with laser wavelengths of 633, 532, and 785 nm.

The literature indicates that surface area is one of the main factors to influence the substance adsorption processes. The N_2_ adsorption isotherms were used to determine the textural parameters, such as specific surface area, pore volume, and pore size. These data were combined with the data from the other samples in each series to facilitate comparison (Figure 7 and Table 4). For the series of magnetite, ME and MR materials, Type II isotherms were observed, which, according to the IUPAC classification, are typically associated with nonporous or macroporous materials.^38^ The specific surface area (*S*_BET_) obtained from the isotherms for the magnetites ME and MR was 37 m^2^ g^–1^ and 69 m^2^ g^–1^, respectively. The synthetic route (rapid for MR versus aged for ME) has resulted in different textural parameters for each magnetite. The rapid route yielded a specific surface area of 86% higher than the aged method. The specific surface area values obtained by the magnetite materials (MR and ME) are in agreement with those ones obtained in the literature.^31–41^

The hysteresis observed in both samples is classified as type H3 (IUPAC), indicating the presence of mesopores formed by the close aggregation of iron nanoparticles. This feature can enhance adsorption processes, given that surface area is a key factor influencing adsorption efficiency. As can be seen, the sample produced via the fast route (MR sample) exhibits the highest surface area of 69 m^2^ g^–1^, which is expected to achieve superior adsorption performance.

### 3.2 Characterization of Cr-Doped Magnetic Nanoparticles

The chemical composition of the samples of Cr-doped magnetite nanoparticles obtained by atomic absorption analyses showed the presence of Cr (Figure 4). We can observe that increasing the amount of CrCl_3_ in the medium increases the Cr content in magnetite, as expected. As can be seen, increasing the aging time and temperature may lead to an increase in Cr concentration in the magnetite lattice. It has been previously reported that aging can influence the final catalyst properties, such as surface area, porosity, and catalyst composition.^42^

**Figure 4:**
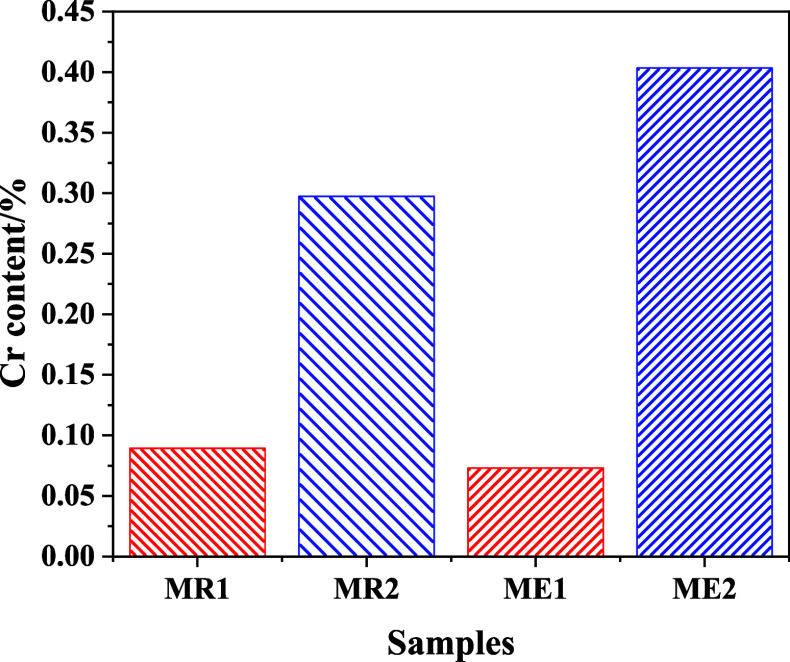
Cr content in samples Cr-doped.

A comparison of the FTIR spectra of Cr-undoped ME and MR samples with those of MR1 and ME1, containing low Cr^3+^ doping, and MR2 and ME2, exhibiting higher Cr^3+^ doping, reveals clear and progressive modifications in the metal–oxygen vibrational region (*ca.* 400–700 cm^–1^), which is consistent with the incorporation of Cr^3+^ into the magnetite lattice (Figure 5).

**Figure 5:**
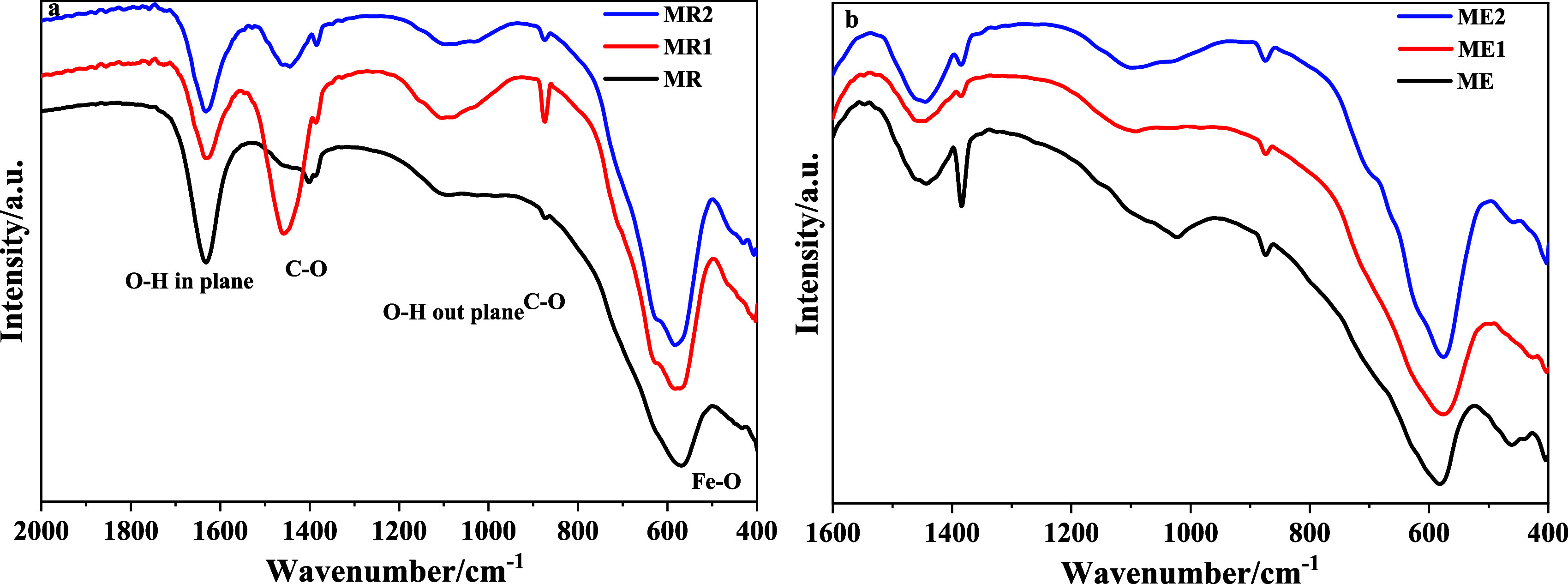
FTIR spectra of (a) MR, MR1, and MR2 samples and (b) ME, ME1, and ME2 samples.

The characteristic Fe–O stretching band of magnetite, typically observed near 570–600 cm^–1^, becomes progressively more intense and slightly shifted when moving from the lower Cr^3+^-doped samples (MR1 and ME1) to the higher chromium-containing samples (MR2 and ME2). This behavior is expected when Cr^3+^ replaces Fe^3+^ in octahedral sites, since the substitution alters the local metal–oxygen bonding environment. Chromium has a different ionic radius and forms slightly different M–O bond strengths compared to Fe^3+^, which can lead to subtle variations in band position, bandwidth, and intensity. The gradual enhancement of this band with increasing Cr content strongly suggests successful lattice incorporation rather than simple surface adsorption of chromium species.

In the low-wavenumber region (≈400–480 cm^–1^), subtle but systematic variations in the Fe–O vibrational band are observed across all samples (MR, MR1, MR2, ME, ME1, and ME2), reflecting differences between the undoped magnetite and those containing low (MR1 and ME1) and higher Cr^3+^ contents (MR2 and ME2). These changes are most likely associated with the partial substitution of Fe^3+^ by Cr^3+^ ions at octahedral sites of the inverse spinel lattice. Since Cr^3+^ has a slightly smaller ionic radius and different metal–oxygen bond strength compared to Fe^3+^, its incorporation may induce local modifications in the octahedral M–O bond environment, leading to small changes in band shape and relative intensity. However, the absence of significant band shifts or pronounced broadening indicates that the overall spinel framework is preserved and that chromium incorporation produces only localized structural perturbations within the Fe_3_O_4_ lattice, as typically observed for low to moderate dopant concentrations.

These spectral changes are consistent with a progressive modification of the spinel structure as Cr^3+^ content increases, reflecting the well-documented overlap of Fe–O and Cr–O vibrational modes. The FTIR analysis therefore corroborates that chromium was effectively incorporated into the magnetite lattice, with the extent of the band modifications correlating with the Cr^3+^ concentration in the samples (MR < MR1 < MR2, and ME < ME1 < ME2).

The structural Rietveld refinement of the powder XRD patterns (Figure 6) was performed by fitting *pseudo*-Voigt functions (Fd3m space group for magnetite). The quality of the Rietveld refinement is listed in Table 3. Analyzing these results, we can observe that the lattice parameters changed upon insertion of Cr. This can be explained by the difference in the diameters of Fe^3+^ (ionic radius, 65 pm) and Fe^2+^ (78 pm) being replaced by Cr^3+^ (61 pm), indicating that a substitution of iron ions by chromium occurred.

**Figure 6:**
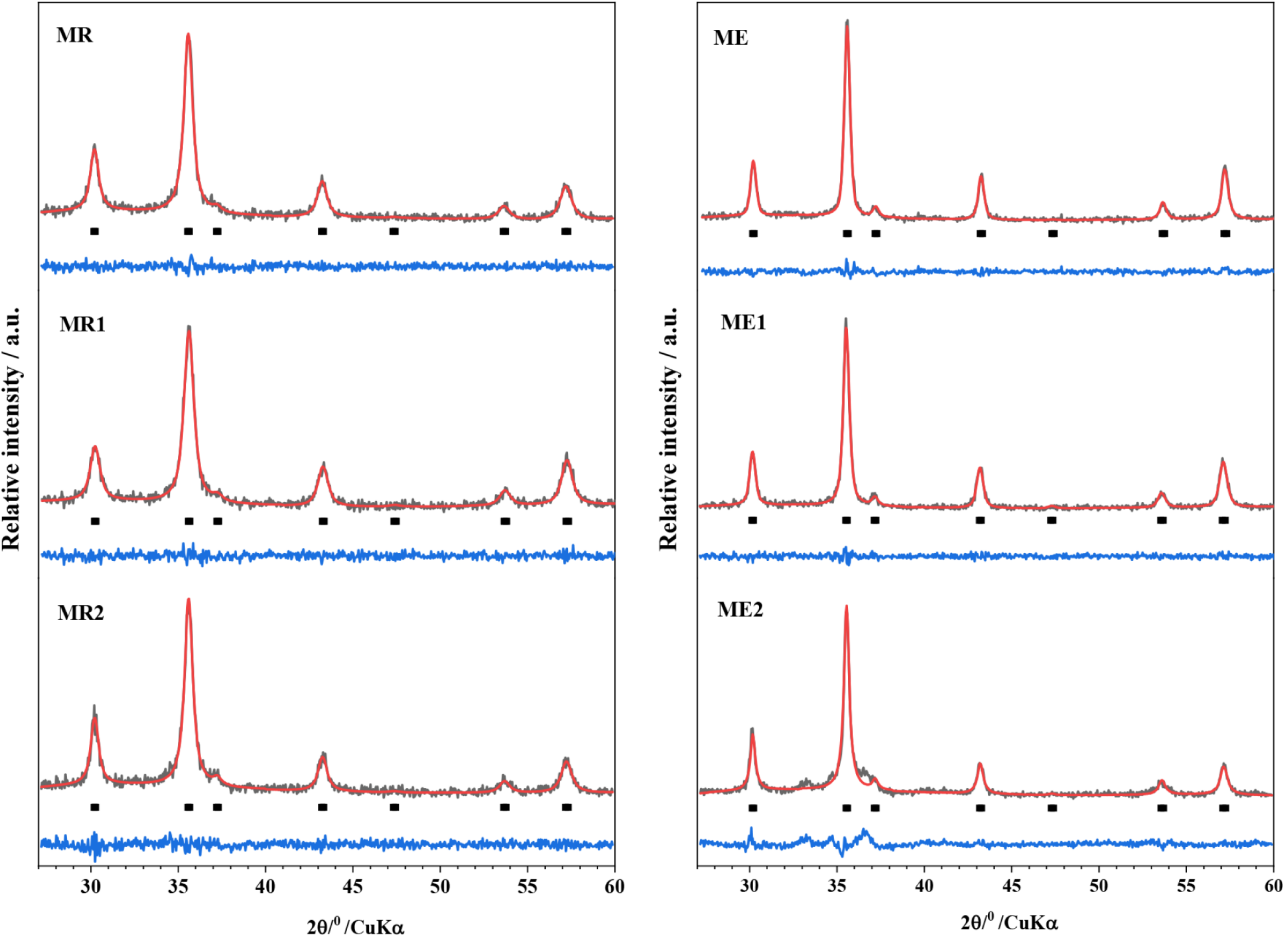
Powder X-ray diffraction patterns (black) of the (left): MR, MR1, and MR2; and (right): ME, ME1, and ME2. The solid (red) lines correspond to the profiles fitted through Rietveld refinement.

**Table 3:** Structural Parameters and Rietveld Agreement Factors for MR and ME Samples

	Samples					
Parameter	MR	MR1	MR2	ME	ME1	ME2
a = b = c	8.369 ± 0.003	8.360 ± 0.002	8.365 ± 0.004	8.367 ± 0.001	8.379 ± 0.001	8.375 ± 0.003
*R*_p_	7.71	6.82	8.95	6.25	7.15	10.9
*R*_exp_	10.3	9.12	11.7	8.22	9.27	14.8
*S*	0.99	0.99	1.00	0.84	0.94	1.38
*R*_B_	2.94	2.71	4.18	2.71	2.72	7.79
*R*_F_	2.47	2.04	3.41	2.53	2.26	4.54

The systematic changes detected in the FTIR metal–oxygen vibrational bands with increasing Cr^3+^ content correlate well with the Rietveld refinement results, which reveal subtle variations in the lattice parameter but no phase transformation, confirming that chromium incorporation produces localized structural perturbations within the magnetite lattice rather than long-range structural changes.

The surface chemical composition and oxidation states of the synthesized magnetite nanoparticles, both Cr-undoped (MR and ME) and Cr-doped (MR1, MR2, ME1, and ME2), were investigated by X-ray photoelectron spectroscopy (XPS). This technique provides surface-sensitive information and is therefore particularly relevant for understanding adsorption phenomena, which are governed by interfacial chemical states rather than the bulk structure.

The XPS survey spectra of the undoped samples (MR and ME) confirm the presence of Fe and O as the main surface elements, with no detectable signals from contaminants or secondary phases, indicating the high chemical purity of the materials (Figure 7). In the Cr-doped samples, additional weak signals corresponding to chromium were detected, which is consistent with the intentionally low Cr content incorporated into the magnetite structure, as previously confirmed by atomic absorption analysis (see Tables S1–S6 in the Supporting Information). The low atomic percentages of Cr observed by XPS are therefore expected and indicate that chromium is present as a minor dopant rather than as a segregated surface phase.

**Figure 7:**
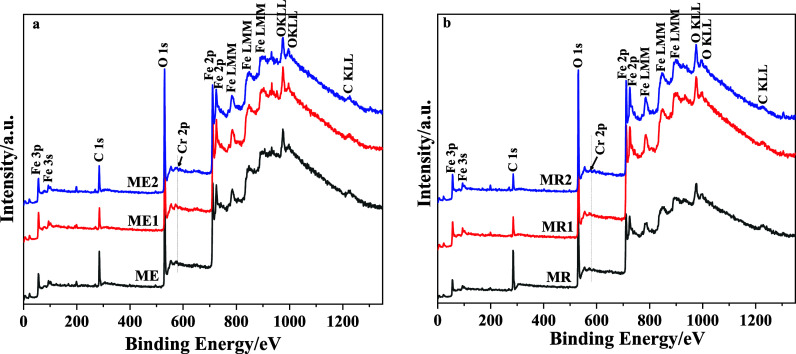
Survey X-ray photoelectron spectroscopy spectra of the samples: (a) ME, ME1, and ME2, and (b) MR, MR1, and MR2.

The high-resolution Fe 2p spectra of all samples exhibit the characteristic doublet corresponding to Fe 2p_3/2_ and Fe 2p_1/2_, with binding energies centered around ∼710.5–711.5 eV and ∼724.0–725.0 eV, respectively (Figure 8). These values are typical of iron oxides with mixed Fe^2+^/Fe^3+^ valence states, as expected for magnetite or partially oxidized magnetite.^43^ The presence of broad Fe 2p peaks and the absence of intense satellite features at higher binding energies indicate that Fe^3+^ is predominantly incorporated within the spinel structure rather than forming separate Fe_2_O_3_ phases at the surface.^43^

**Figure 8:**
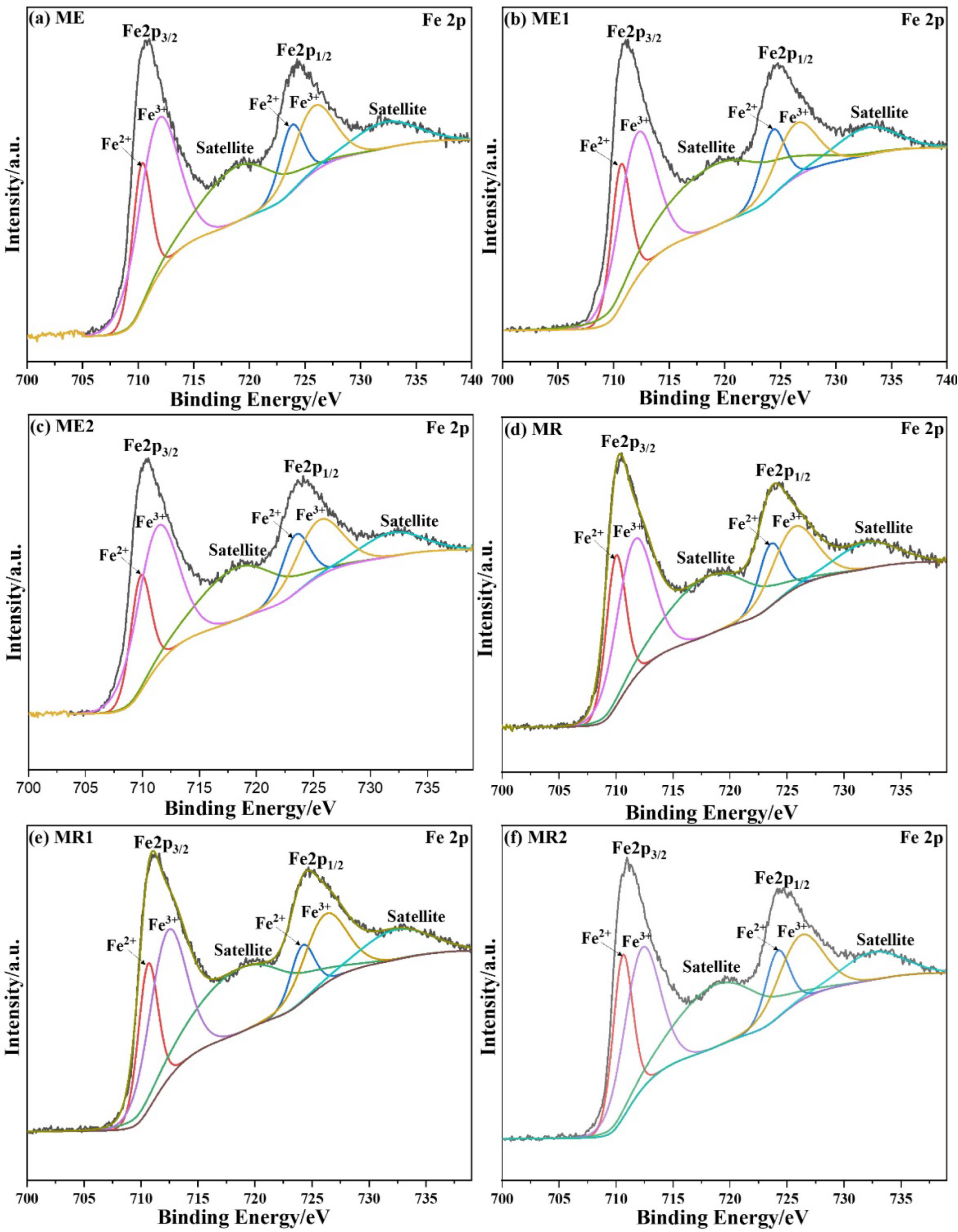
High-resolution XPS spectra in the Fe 2p region of the samples: (a) ME, (b) ME1, (c) ME2, (d) MR, (e) MR1, and (f) MR2.

Importantly, no systematic shift in the Fe 2p binding energies was observed upon Cr incorporation, indicating that chromium doping does not significantly alter the average oxidation state of the surface iron species. This observation is consistent with the XRD and FTIR results, which showed the preservation of the magnetite spinel structure upon Cr substitution.

The high-resolution O 1s spectra of all samples can be consistently deconvoluted into two main contributions: a lower binding energy component at ∼529.4–530.3 eV, attributed to lattice oxygen (O^2–^) in metal–oxygen bonds, and a higher binding energy component at ∼531.2–531.5 eV, commonly associated with surface hydroxyl groups, adsorbed water, and/or oxygen species in more distorted or defective coordination environments (Figure S1).

Quantitative analysis of the fitted peak areas shows that the high-binding-energy O 1s contribution is already significant in the undoped materials and does not exhibit a systematic increase upon chromium incorporation. In the ME series, only minor and sample-dependent variations in the relative contribution of this component are observed, whereas in the MR series, a progressive decrease in the fraction of the higher binding energy component is evident from MR to MR2, accompanied by a relative increase in the lattice-oxygen contribution. These results indicate that chromium incorporation does not uniformly enhance surface hydroxylation but rather induces sample-dependent modifications in the oxygen chemical environment, leading to a redistribution between lattice and nonlattice oxygen species at the surface.

Although chromium is present at low concentrations, the Cr 2p region could be clearly identified in the doped samples (Figure S2). The Cr 2p_3/2_ peak appears at binding energies around ∼577.0–577.8 eV, which is characteristic of Cr^3+^ species, typically associated with Cr_2_O_3_-like coordination environments. No peaks corresponding to Cr­(VI) species (which would appear at significantly higher binding energies, ∼579–580 eV) were detected. This confirms that chromium remains in the trivalent state after synthesis and during handling and does not undergo oxidation at the surface.^44^

The absence of additional Cr-related satellite features or distinct oxide phases further supports the conclusion that Cr^3+^ is incorporated into the magnetite lattice or strongly associated with the surface in a highly dispersed manner rather than forming segregated chromium oxide domains.

X-ray diffraction patterns combined with Rietveld refinement confirmed that all samples, both doped and undoped, retained the spinel structure of magnetite after contact with KMnO_4_. No additional reflections associated with secondary phases were detected. In particular, diffraction peaks characteristic of Cr_2_O_3_ (2θ ≈ 24.5°, 33.6°, and 36.3°) or crystalline chromate phases (2θ ≈ 20–21° and 29–34°) were not observed, indicating the absence of segregated chromium oxide or chromate phases.^45,46^

The refined lattice parameters exhibited only minor and systematic variations following the adsorption experiments (MR: 8.369 → 8.358 Å; MR1: 8.360 → 8.357 Å; MR2: 8.365 → 8.350 Å; ME: 8.367 → 8.365 Å; ME1: 8.379 → 8.365 Å; ME2: 8.375 → 8.365 Å) (see Figure S3 and Table S7 in the Supporting Information). All values remained within the characteristic range reported for magnetite (8.38 Å ≥ *a*_0_ ≥ 8.34 Å), whereas maghemite typically exhibits a smaller lattice parameter of approximately 8.34 Å.^33,37^ The slight unit cell contraction observed after adsorption is therefore attributed to adsorption-induced lattice relaxation or limited surface oxidation effects rather than to a structural phase transformation.^33,47^

In addition to the solid-state structural analysis, the possible influence of the pH and redox processes in solution may be considered. The adsorption experiments were conducted under controlled pH conditions, which are known to influence the speciation and redox behavior of both Mn and Cr. Under these conditions, no evidence of Cr­(III) oxidation to soluble Cr­(VI) species was observed. The formation of chromate ions would be expected to produce characteristic absorption bands in the UV–vis spectra, which were not detected during the experiments. This indicates that chromium remained in the trivalent state within the magnetite lattice and did not participate in solution-phase redox processes even in the presence of permanganate.

The incorporation of Cr into magnetite nanoparticles maintained the N_2_ adsorption isotherms in the type II profile, similar to that observed in Cr-undoped nanoparticles. However, the addition of Cr enhances the textural parameters, such as specific surface area (*S*_BET_), total pore volume (*V*_total_), and mesopore volume (*V*_meso_), in both synthetic routes (Figure 9 and Table 4).

**Figure 9:**
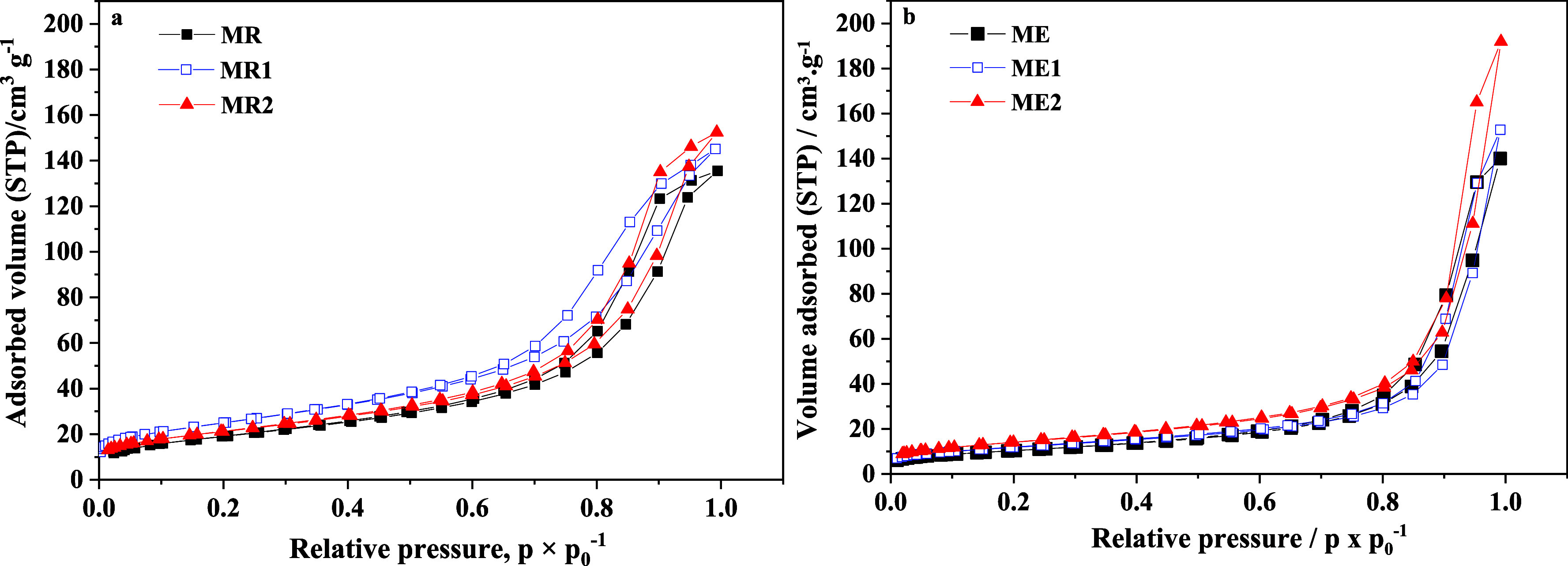
N_2_ adsorption isotherms for: (a) MR, MR1, and MR2 samples and (b) ME, ME1, and ME2 samples.

**Table 4:** Textural Parameters Obtained for the ME and MR Materials, Cr-Doped and Undoped^a^

Sample	*S*_BET_/m^2^ g^–1^	*V*_total_/cm^3^ g^–1^	*V*_meso_/cm^3^ g^–1^	P_D_/nm
**ME**	37	0.216	0.216	16.89
**ME1**	42	0.237	0.231	29.04
**ME2**	51	0.297	0.292	29.06
**MR**	69	0.209	0.204	12.24
**MR1**	90	0.224	0.214	9.498
**MR2**	76	0.236	0.227	12.24

a *S*_BET_: specific surface area calculated by BET method (m^2^ g^–1^). *V*_total_: total pore volume obtained at p/p_0_ = 0.99 (m^3^ g^–1^). *V*_meso_: mesopore volume obtained by BJH (cm^3^ g^–1^). P_D_: pore size diameter obtained by BJH (nm).

In the aged magnetite materials (ME, ME1, and ME2 samples), the sample with the highest Cr content (ME2) exhibited a 38% increase in specific surface area and total mesopore volume, along with an 82% increase in average pore size, attributed to the slow deposition of Cr^3+^ during the aging process (Table 4). Also, in the rapidly produced magnetite (MR, MR1, and MR2 samples), the Cr doping increased the specific surface area. As observed in Table 4, the surface of sample MR2 is smaller than the surface of sample MR1. Probably due to the faster particle aggregation in MR2 allowed by the improved electrostatic interaction caused by a high amount of Cr^3+^ ions, it could contribute to a decrease in the specific surface area,^48^ resulting in different particle sizes and shapes as a function of Cr doping, as well as improving the textural parameters. Also, the Cr doping strategy can modify the surface chemistry of magnetite-based materials, making them promising materials for the adsorption of ionic species.

### 3.3 Kinetic Curves and Adsorption Isotherms

Figure 10 illustrates the adsorption of MnO_4_^–^ by Cr-doped and undoped magnetite nanoparticles over time, at an initial MnO_4_^–^ concentration of 50 mg L^–1^ and a sorbent concentration of 1 g L^–1^. The adsorption kinetics were analyzed to gain deeper insight into the adsorption rate and mechanism of the sorption process on the adsorbent surface and to identify a model capable of predicting the adsorption rate over time, a critical factor in the treatment of aqueous effluents.^49^ For this, two adsorption kinetic models—*pseudo*-first-order and *pseudo*-second-order—were applied. The experimental data fitted with the *pseudo*-first-order and *pseudo*-second-order models are presented as curves in Figure 10a, b. The straight lines fitted to the experimental data by the intraparticle diffusion model are shown in Figure 11a, b. The kinetic parameters derived from the *pseudo*-first-order and *pseudo*-second-order models are summarized in Table 5, while those obtained from the intraparticle diffusion model are listed in Table 6.

**Figure 10:**
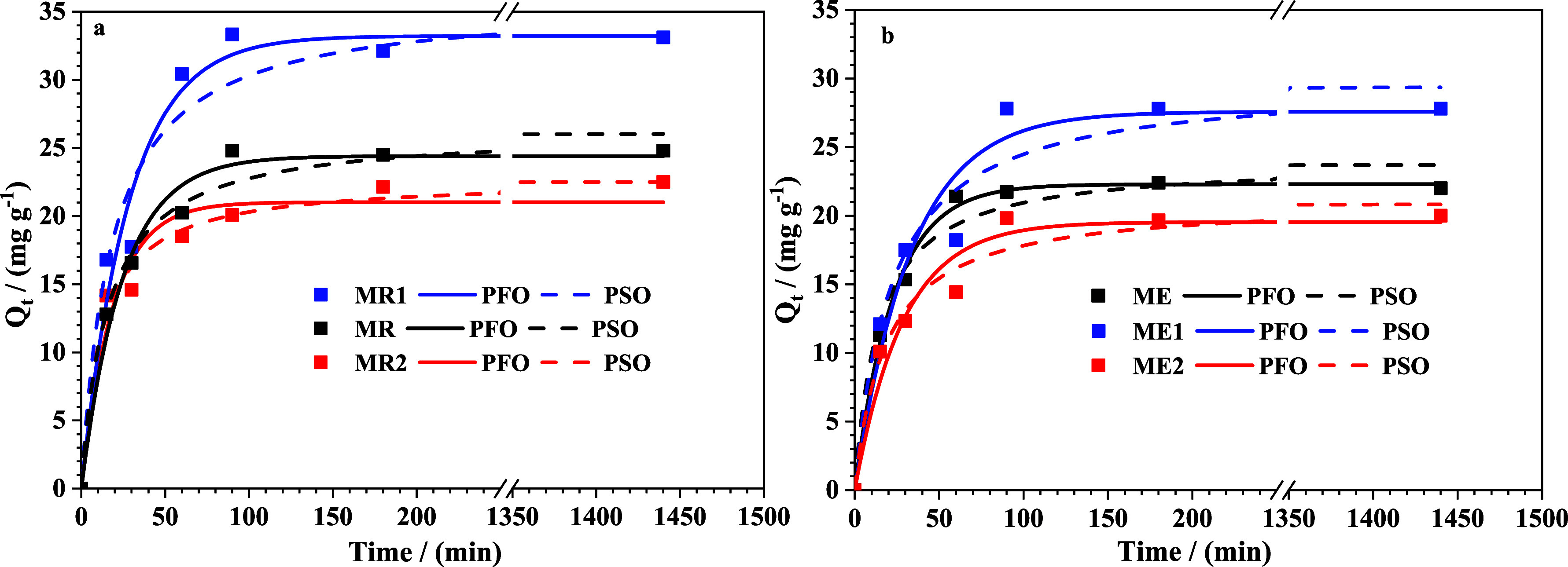
Adsorption capacity (*Q_t_*) of MnO_4_^–^ on the samples: (a) MR, MR1, and MR2 and (b) ME, ME1, and ME2, as a function of time (*t*) at 25 °C, with the *pseudo*-first and *pseudo*-second order curves fitted to the experimental data and (c) and (d) intraparticle diffusion plots for the adsorption of MnO_4_^–^ on the same samples.

**Figure 11:**
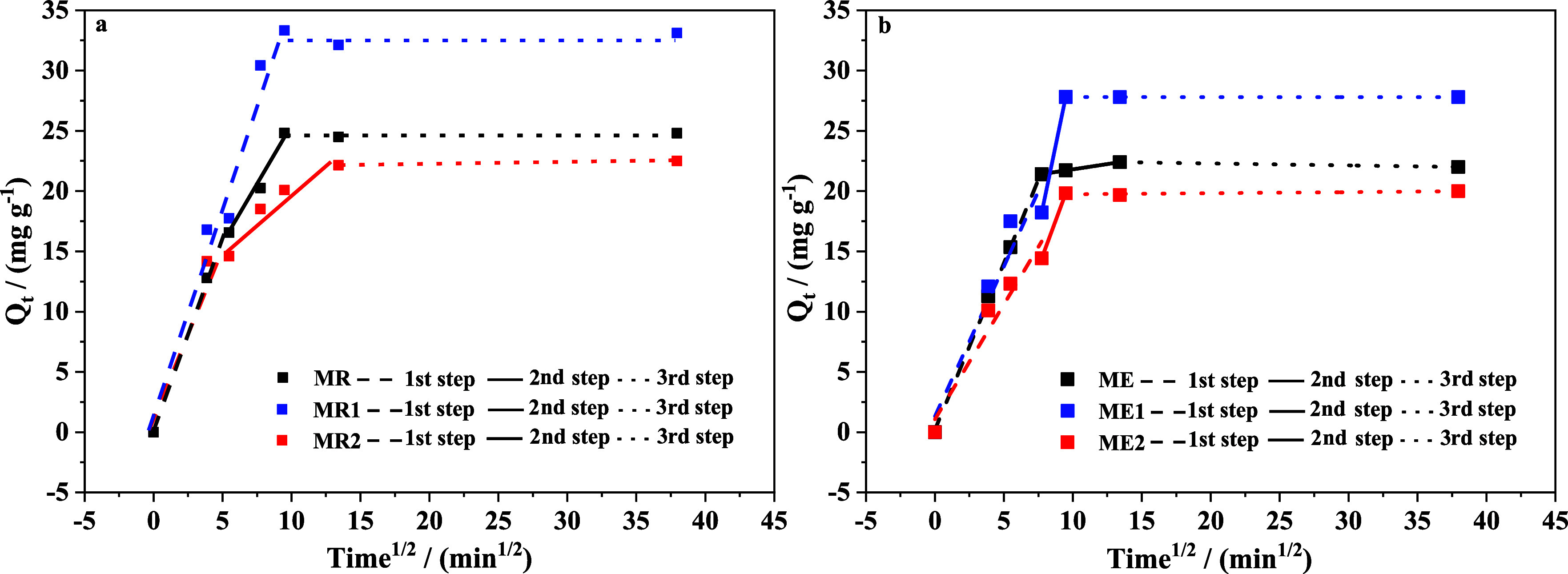
Intraparticle diffusion plots for the adsorption of MnO_4_^–^ on (a) MR, MR1, and MR2 samples and (b) ME, ME1, and ME2 samples.

**Table 5:** Kinetic Parameters Estimated by the *Pseudo*-First and *Pseudo*-Second Order Models for Adsorption of MnO_4_^–^ on MR, MR1, MR2, ME, ME1, and ME2 Samples

Sample	*Q*_*e*_/mg g^–1^	*K*/min^–1^	$R_{adj}^{2}$	$\chi_{red}^{2}$
*Pseudo*-first order				
MR	24.41 ± 0.77	0.040 ± 0.005	0.9795	1.69
MR1	33.23 ± 1.53	0.035 ± 0.006	0.9614	6.05
MR2	21.02 ± 1.05	0.053 ± 0.012	0.9397	3.66
ME	22.29 ± 0.35	0.044 ± 0.003	0.9947	0.37
ME1	27.58 ± 1.69	0.030 ± 0.006	0.9380	6.65
ME2	19.54 ± 1.02	0.035 ± 0.007	0.9487	2.70
*Pseudo*-second order				

MR	26.31 ± 0.98	0.0024 ± 0.0005	0.9799	1.65
MR1	35.77 ± 2.41	0.0015 ± 0.0006	0.9395	9.50
MR2	22.69 ± 0.79	0.0037 ± 0.0008	0.9804	1.19
ME	23.93 ± 1.06	0.0029 ± 0.0008	0.9710	2.02
ME1	29.79 ± 2.06	0.0015 ± 0.0005	0.9407	6.36
ME2	21.08 ± 1.12	0.0026 ± 0.0007	0.9613	2.04

**Table 6:** Kinetic Parameters Estimated by the Intraparticle Diffusion Model for Adsorption of MnO_4_^–^ on MR, MR1, MR2, ME, ME1, and ME2 Samples

	*K*_1_	*K*_2_	*K*_3_	$R_{i,1,adj}^{2}$	$R_{i,2,adj}^{2}$	$R_{i,3,adj}^{2}$
MR	3.075	2.035	0.004	0.9903	0.9632	0.7374
MR1	3.569	-	0.009	0.9662	-	0.8634
MR2	2.849	0.906	0.009	0.8643	0.8584	0.8934
ME	2.764	0.175	0.016	0.9987	0.9998	1
ME1	2.488	5.511	2.290	0.8933	1	0.7142
ME2	1.905	3.097	0.009	0.9205	1	0.3719

As can be seen in Figure 10, all the samples (doped samples and undoped) reached adsorption capacity equilibrium within 100 min. The *pseudo*-second order model fitted the experimental data better than the *pseudo*-first order model (values of $R_{adj}^{2}$ and $\chi_{red}^{2}$ ). In general, the *pseudo*-second-order kinetic model assumes that the adsorption process occurs on localized sites with no interaction between adsorbates, and maximum adsorption corresponds to a saturated monolayer of adsorbates onto the adsorbent surface. Furthermore, it could be indicated that the adsorption process was mainly a chemisorption process, and the exchange of electrons or electrostatic forces between adsorbents and MnO_4_^–^ was involved in the process.^50^ The samples MR1 and ME1 exhibited higher efficiency and adsorption capacity compared to the other tested samples, while the samples MR2 and ME2 showed lower efficiency. Considering that samples MR1 and ME1 present quite different specific surface areas, we suggest that in this experimental condition, the surface area could be less important to adsorption of MnO_4_^–^ ions compared with the amount of chromium in magnetite, *ca.* 0.1%. This composition should provide a good superficial condition on Cr-doped magnetite to allow the permanganate adsorption.

In Figure 11, the adsorption of MnO_4_^–^ on doped samples and undoped samples occurred in two or more steps: the initial phase of rapid adsorption within the first 15 min, region I, is attributed to the external surface adsorption or instantaneous physical adsorption; the intermediate portion up to 100 min, region II, is the gradual adsorption step, where intraparticle diffusion is controlled and corresponds to porosity-dependent adsorption; and the extended portion, region III, is the final equilibrium step. Similar observations have been made on the adsorption of anionic azo dye (Congo Red) on Fe_3_O_4_ nanoparticles loaded with papaya^51^ and lead on maghemite nanoparticles.^52^ The slope of each linear portion is denoted by *k*_1_, *k*_2_, and *k*_3_ (Table 6) corresponding to the rate constants of regions I, II, and III, respectively.

The samples synthesized by the rapid method, Cr-doped or undoped (MR, MR1, and MR2), exhibited a larger surface area than that of the aged samples (ME, ME1, and ME2). This promotes faster kinetics because of the increased availability of adsorption sites. This is manifested in the higher *k*_1_ of samples MR, MR1, and MR2 as compared with the samples ME, ME1, and ME2. On the other hand, *k*_2_ was higher for samples ME1 and ME2, which are the samples with the highest porosity, reinforcing the porosity dependence of the uptake kinetics in this intermediate region. Moreover, these samples are the ones that exhibit the largest pore diameter, and diffusion is faster through the pores and it is retarded when the pore is small.^53^ The last step involved the final equilibrium step where the intraparticle diffusion starts to slow down due to saturation.^54^

### 3.4 Adsorption Isotherms

To investigate the influence of Cr^3+^ doping in magnetite on adsorption capacity for MnO_4_^–^ by Cr-doped and undoped magnetite nanoparticles, several adsorption experiments were performed. The adsorption isotherms were carried out at room temperature, with an equilibrium time of 180 min and an agitation speed of 180 rpm. An adsorption isotherm describes the distribution of adsorbate molecules between the liquid and solid phases when equilibrium is reached during the adsorption process. Adsorption isotherms provide valuable information about adsorption capacity, binding affinity, and the surface properties of the adsorbent, elucidating the interaction mechanism between the adsorbate and the adsorbent. Analyzing the isotherm data by fitting it to various models is a crucial step in identifying the most appropriate model for design purposes.^55^

The adsorption behavior was analyzed by using two well-established isotherm models: Langmuir and Freundlich. The Langmuir isotherm assumes monolayer adsorption on a surface with a finite number of uniform adsorption sites, without lateral movement of the adsorbate across the surface.^56^ The Freundlich isotherm model, on the other hand, assumes a surface with heterogeneous energy sites, where the energy term in the Langmuir equation changes with the surface coverage.^56^ The experimental data in Figure 12 were fitted to the Langmuir and Freundlich models, and the corresponding results are presented in Table 7.

**Figure 12:**
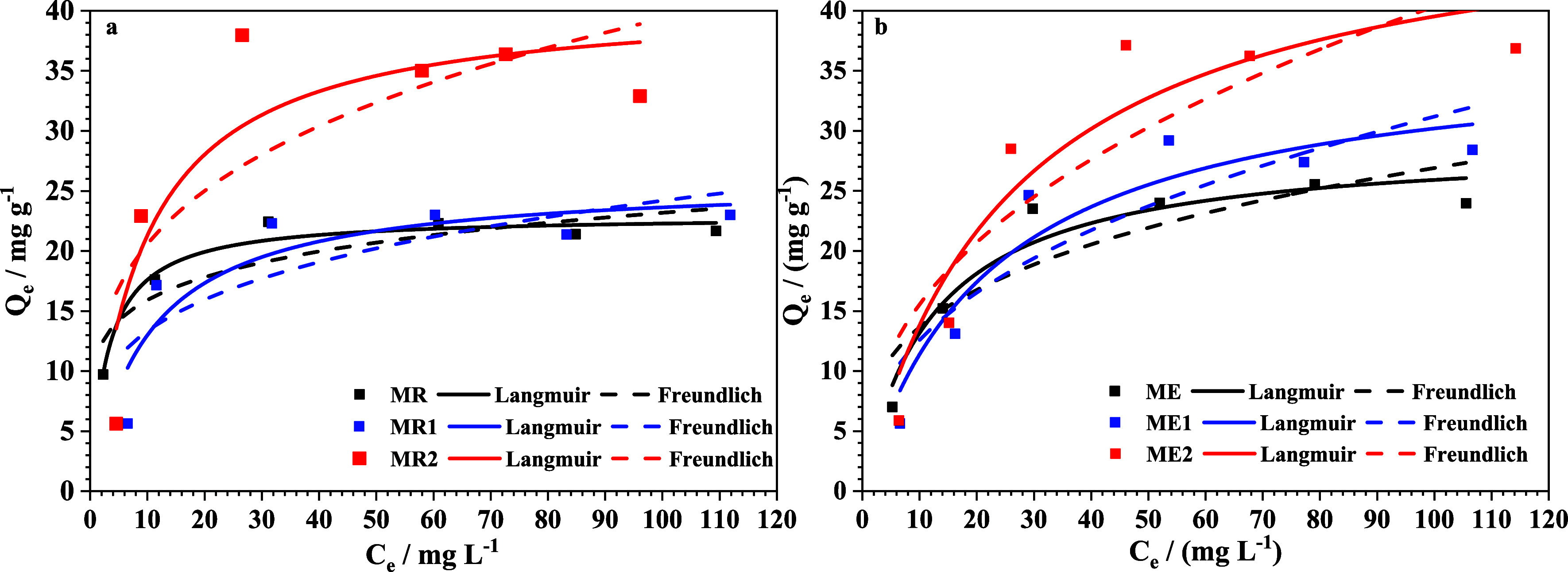
Equilibrium adsorption isotherm of MnO_4_^–^ onto (a) MR, MR1, and MR2 samples and (b) ME, ME1, and ME2 samples.

**Table 7:** Estimated Values for Constants for the Langmuir and Freundlich Models at Different Concentrations of Samples of Cr-Doped Magnetite Nanoparticles and pH 5.6

	Langmuir			
Sample	*Q*_*max*_ (mg g^–1^)	*K*_*L*_ (L mg^–1^)	$R_{adj}^{2}$	$\chi_{red}^{2}$
MR	22.96 ± 0.59	0.33 ± 0.06	0.96	0.94
MR1	25.99 ± 2.93	0.10 ± 0.05	0.77	10.69
MR2	40.98 ± 5.47	0.11 ± 0.06	0.76	37.03
ME	29.04 ± 2.09	0.08 ± 0.02	0.92	4.03
ME1	36.99 ± 5.03	0.04 ± 0.02	0.88	10.88
ME2	34.77 ± 3.83	0.05 ± 0.02	0.91	7.04

	Freudlich			
	*K*_*f*_	*n*	$R_{adj}^{2}$	$\chi_{red}^{2}$
MR	10.92 ± 2.15	6.12 ± 1.88	0.74	6.51
MR1	7.34 ± 3.30	3.86 ± 1.67	0.57	19.75
MR2	10.56 ± 0.12	4.30 ± 0.04	0.93	1.85
ME	6.93 ± 2.55	3.40 ± 1.06	0.74	13.74
ME1	5.07 ± 2.62	2.53 ± 0.81	0.74	24.08
ME2	5.07 ± 2.36	2.60 ± 0.77	0.78	18.12

Based on the comparison of the *R* ^2^ and $\chi_{red}^{2}$ values (Table 7), Langmuir gave a better fit than Freundlich to the adsorption isotherms of MnO_4_^–^, suggesting that MnO_4_^–^ adsorption mainly belonged to monolayer adsorption.^57,58^ Also, it can be attributed to the strong electrostatic interaction between the positive charges on the ferrite nanoparticles’ surface and the negative charges of MnO_4_^–^.^59^ Thus, it may be suggested that the adsorption could be site-specific and thus a monolayer is formed. An exception to this behavior is the MR2 sample, which was better fitted to the Freundlich model. Unlike the Langmuir isotherm model, the Freundlich model is not restricted to monolayer formation and can also be applied to multilayer adsorption. The Freundlich isotherm model expression defines the heterogeneity of the surface, as well as the exponential distribution of the active sites and the active sites energies.

It is observed that when the Cr concentration increases in the magnetite nanoparticles, the *Q*_*max*_ also increases (Table 7). This indicates that the doping of the magnetite with Cr may increase the adsorption efficiency, since the Cr^3+^ also has an affinity for the MnO_4_^–^ by electrostatic attraction. In addition, it is worthy to mention that the surface area of the two sets of samples (fast synthesis and slow synthesis) increases in the following order: ME < ME1 < ME2 and MR < MR2 < MR1, which agrees with the order of *Q*_*max*_ increment. This was expected, given that a larger surface area could provide more active adsorption sites.^60^ The fact that the MR2 sample did not exhibit the highest surface area within its sample set may suggest the formation of Cr oxide, which could lead to a reduced surface area.

Comparing the samples synthesized by the fast method (MR) with those synthesized by the aging method (ME), we can observe that the samples synthesized by the aging method exhibited a higher *Q*_*max*_. Since surface area and pore size are the key factors that greatly affect the adsorption phenomenon,^53^ we can say that with our samples, the most important factor was the pore size. The sample that showed the highest *Q*_*max*_ is MR2, which, as previously mentioned, follows the Freundlich model, which does not predict the formation of a monolayer. In this case, the value of 1/*n* is important, since it represents the intensity of the adsorption or surface heterogeneity, indicating the energy relative distribution and the heterogeneity of the adsorbate site. When 1/*n* is greater than zero (0 < 1/*n* < 1), the adsorption is favorable; when 1/*n* is greater than 1, the adsorption process is unfavorable; and it is irreversible when 1/*n* = 1. For the sample MR2, 1/*n* = 0.23, which indicates that the adsorption is favorable.

In addition to these observations, it is important to emphasize that, although the chromium content in the doped samples is very low (≤0.4 wt %), even such minor levels of substitution can subtly modify the surface chemistry of magnetite in ways that affect the mechanism of MnO_4_^–^ adsorption. Cr^3+^ incorporation is expected to slightly increase the density of surface hydroxyl groups and to alter the local charge distribution at Fe–O sites, thereby enhancing the electrostatic attraction between the negatively charged MnO_4_^–^ ions and the nanoparticle surface. These modifications are not sufficiently pronounced to produce detectable changes in bulk-sensitive structural characterization techniques such as FTIR or XRD. However, they are sufficient to influence adsorption behavior, particularly when combined with the observed increase in surface area. Consequently, the higher *Q*_*max*_ values obtained for the Cr-doped samples likely result from a synergistic contribution of two factors: (i) a modest but meaningful increase in accessible active sites associated with changes in surface area and texture, and (ii) a slight enhancement in surface affinity for MnO_4_^–^ arising from Cr-induced modifications in the local chemical environment. This interpretation defines the proposed adsorption mechanism and is consistent with the progressive improvement in adsorption performance observed across the doped series, supporting the conclusion that Cr incorporation, even at very low concentrations, contributes positively to MnO_4_^–^ removal.

Selective oxyanion adsorption in complex aqueous matrices is primarily controlled by interfacial charge, surface coordination chemistry, and the redox properties of iron oxides, as discussed in several reviews on magnetic nanomaterials for water purification.^61–63^ Fe_3_O_4_-based adsorbents have been shown to preferentially interact with high-valence, strongly oxidizing oxyanions such as MnO_4_^–^ and CrO_4_^2–^, owing to electrostatic attraction under acidic to neutral conditions and the involvement of surface Fe^2+^/Fe^3+^ sites in partial electron-transfer interactions. In multicomponent systems, weakly interacting anions (for example Cl^–^ and NO_3_^–^) generally exert minimal competition, whereas polyatomic species with higher charge density or stronger hydration, including SO_4_^2–^, HCO_3_^–^, PO_4_^3–^, can suppress uptake by occupying active sites or altering the surface potential. Moreover, metal doping, particularly with trivalent cations, such as Cr^3+^, has been reported to modify surface acidity and Lewis acidity, thereby enhancing selectivity toward oxidizing oxyanions through increased site heterogeneity and tuning of the local electronic environment.

Although these mechanistic trends support the plausibility of selective MnO_4_^–^ adsorption by Cr-doped magnetite in more complex matrices, evaluating competitive adsorption or performance in real mining effluents was beyond the scope of this study, which focused on elucidating the intrinsic adsorption behavior of the doped materials under controlled, single-solute conditions. Nevertheless, the insights presented here provide a foundation for future studies addressing the effects of coexisting ions and matrix complexity to assess the selectivity and environmental robustness of these materials.

## 4 Final Considerations

In summary, our findings highlight the effectiveness of Cr-doped magnetite nanoparticles as an innovative and effective adsorbent for the removal of permanganate ions (MnO_4_^–^) from aqueous solutions. The synthesis and characterization results confirm the successful incorporation of Cr into the magnetite structure, which contributes to improvements in structural and textural properties, including surface area and porosity. Adsorption kinetic studies revealed that the pseudo-second-order model provides a good description of the process, suggesting chemisorption as the possible mechanism. In addition, both the Freundlich and Langmuir isotherm models were fitted to the experimental data. The high adsorption capacity of the Cr-magnetite underscores its potential as a sustainable solution for addressing water contamination in regions affected by mining activities. Overall, this class of magnetite-based nanoparticle emerges as a promising material for environmental remediation, contributing to the development of cost-effective and scalable technologies for the removal of toxic pollutants from water systems.

## Supplementary Material



## Notes

Weak signals corresponding to chromium were observed in the Cr-doped magnetite samples, consistent with the intentionally low Cr content incorporated into the structure, as previously confirmed by atomic absorption analysis (Tables S1–S6); refined lattice parameters showed only minor and systematic variations following the adsorption experiments (MR: 8.369 → 8.358 Å; MR1: 8.360 → 8.357 Å; MR2: 8.365 → 8.350 Å; ME: 8.367 → 8.365 Å; ME1: 8.379 → 8.365 Å; ME2: 8.375 → 8.365 Å) (Figure S3 and Table S7) (PDF)
