# Supplementary material for: Chromium-Doped Magnetite Nanoparticles for Permanganate Ion Removal: Advances in Environmental Remediation
Source: ACS Omega. 2026 May 18;11(21):30640–54. doi: 10.1021/acsomega.5c12244 (PMC13234899; doi:10.1021/acsomega.5c12244)
Supplement: Supplementary file 1 [file ao5c12244_si_001.pdf]

Chromium-Doped Magnetite Nanoparticles for Permanganate Ion Removal: Advances in Environmental Remediation

Daniel de Lima Silva[1], Rubens Lucas de Freitas Filho[2], André Esteves Nogueira[3], Juliana da Fonseca Alves[4], Luiz Fernando Cappa de Oliveira[4], Guilherme Jorge Brigolini Silva[5], Rodrigo S. Corrêa[1], Adilson Candido da Silva[1], Ângela Leão Andrade[1]

[1]Departamento de Química, Instituto de Ciências Exatas e Biológicas, Universidade Federal de Ouro Preto (UFOP), CEP 35400-000, Ouro Preto-MG, Brasil.

[2]Departamento de Química, Instituto de Ciências Exatas, Universidade Federal de Minas Gerais (UFMG), CEP 31270-901, Belo Horizonte-MG, Brazil.

[3]Departamento de Química, Divisão de Ciências Fundamentais (IEF), Instituto Tecnológico de Aeronáutica (ITA), CEP 12228-615, São José dos Campos-SP, Brazil.

[4]Núcleo de Espectroscopia e Estrutura Molecular, Departamento de Química, Universidade Federal de Juiz de Fora (UFJF), MG, 36036-900, Brazil.

[5]Departamento de Engenharia Civil, Escola de Minas, Universidade Federal de Ouro Preto (UFOP), CEP 35400-000, Ouro Preto-MG, Brasil.

Table S1. Elemental identification and atomic quantification obtained by XPS for the undoped magnetite sample (MR).

Name	Peak BE	FWHM eV	Area (P) CPS.eV	Atomic %
Fe2s	843.01	1.92	18944.57	3.79
Fe2p Fe2O3	711.27	3.58	270389.68	10.74
Fe2p3	711.05	2.94	124191.52	7.10
Fe3p3	56.20	3.12	61627.08	22.40
O1s	530.95	2.98	330519.29	55.97

Table S2. Elemental identification and atomic quantification obtained by XPS for the undoped magnetite sample (MR1).

Name	Peak BE	FWHM eV	Area (P) CPS.eV	Atomic %
O KL2	995.23	2.62	9185.36	8.96
Fe2p3	711.37	3.12	91509.82	3.22
O1s	531.42	3.77	377372.36	39.37
Fe3s	93.92	0.64	1605.24	0.66
Fe3p	56.77	3.54	54039.23	7.98
Cr2p Cr2O3	577.44	1.93	16569.83	0.44
O1s Cr oxide	531.42	3.77	377372.36	39.37

Table S3. Elemental identification and atomic quantification obtained by XPS for the undoped magnetite sample (MR2).

Name	Peak BE	FWHM eV	Area (P) CPS.eV	Atomic %
Fe2p Fe2O3	711.27	3.41	281583.73	12.28
Fe2p3	711.10	2.96	143163.70	8.98
Cr2s	697.62	0.61	3157.33	0.69
Cr2p CrF3	577.79	2.14	9778.04	0.46
Cr2p Cr2O3	577.79	2.14	9778.04	0.46
Cr2p	577.79	2.14	9778.04	0.46
O1s Cr oxide	530.96	2.94	359167.98	66.75
Fe3s	93.99	0.79	3947.09	2.89
Cr3s	75.41	1.30	7840.22	7.02

Table S4. Elemental identification and atomic quantification obtained by XPS for the undoped magnetite sample (ME).

Name	Peak BE	FWHM eV	Area (P) CPS.eV	Atomic %
Fe2s	844.88	1.77	20677.93	3.12
Fe2p Fe2O3	711.19	3.51	297984.76	8.92
Fe2p	711.19	3.51	297984.76	8.92
Fe2p3	711.02	3.08	155864.01	6.71
Fe3s	93.77	1.25	4301.09	2.16
Fe3p3	56.17	3.24	71632.29	19.62
O1s	530.92	2.96	396219.22	50.55

Table S5. Elemental identification and atomic quantification obtained by XPS for the undoped magnetite sample (ME1).

Name	Peak BE	FWHM eV	Area (P) CPS.eV	Atomic %
Fe LM8	933.69	1.15	1946.57	1.26
Fe2p1	724.05	2.64	26959.73	3.21
Fe2p3	711.00	3.03	134142.61	6.92
O1s	530.94	2.97	356993.98	54.53
Fe3p	56.20	3.30	68050.31	14.71
Fe2p1	723.86	2.18	6457.80	0.77
Fe2p3	710.61	2.60	32753.52	1.69
O1s	530.30	1.70	55408.78	8.46
O1s Cr oxide	530.30	1.70	55408.78	8.46

Table S6. Elemental identification and atomic quantification obtained by XPS for the undoped magnetite sample (ME2).

Name	Peak BE	FWHM eV	Area (P) CPS.eV	Atomic %
Fe2p1	723.70	2.25	5778.94	0.41
Fe2p3	710.51	2.65	29699.13	0.92
O1s	530.28	2.41	70346.70	6.43
O1s Cr oxide	530.28	2.41	70346.70	6.43
Fe2p1	724.10	2.27	24991.53	1.78
Fe2p3	710.96	3.06	122652.26	3.79
Cr2s	695.16	0.87	1829.78	0.20
Cr2p3	577.00	1.97	3362.72	0.12
O1s	531.02	3.41	390675.35	35.75
O1s Cr oxide	531.02	3.41	390675.35	35.75
Fe3p	56.11	3.19	65123.36	8.43

Table S7. Structural parameters and Rietveld agreement factors for MR and ME samples after contact with $KMnO_4$.

Parameter	Samples					
	MR	MR1	MR2	ME	ME1	ME2
a = b = c	8.358 ± 0.003	8.357 ± 0.002	8.350 ± 0.003	8.365 ± 0.002	8.365 ± 0.002	8.365 ± 0.001
R_p	2.15	2.20	2.42	2.63	2.32	2.34
R_{exp}	2.58	2.74	3.01	3.24	2.86	2.81
S	1.04	1.01	1.01	1.02	1.02	1.05
R_B	2.51	1.59	3.88	5.36	1.80	3.57
R_F	3.69	2.04	4.07	6.48	1.,90	3.62

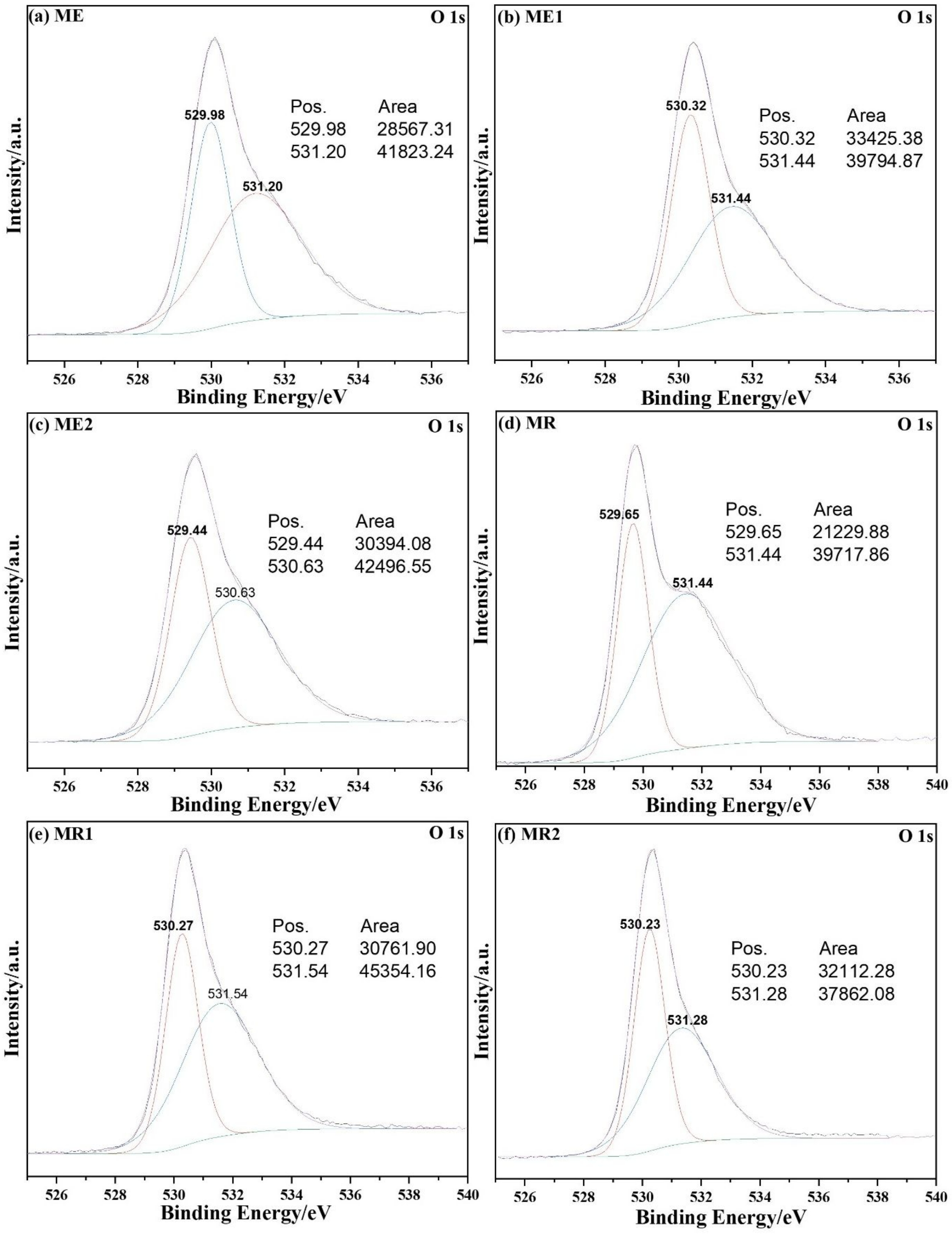

Figure S1. High-resolution XPS spectra in the O 1s region of the samples: (a) ME, (b) ME1, (c) ME2, (d) MR, (e) MR1, and (f) MR2.

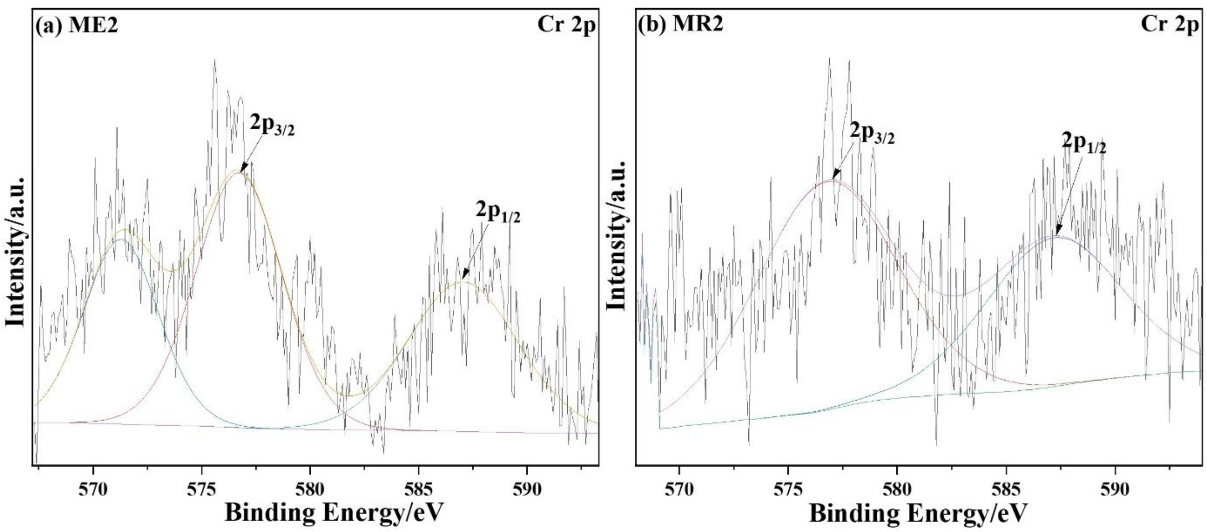

Figure S2. High-resolution XPS spectra in the Cr 2p region of the samples: (a) ME2 and (b) MR2.

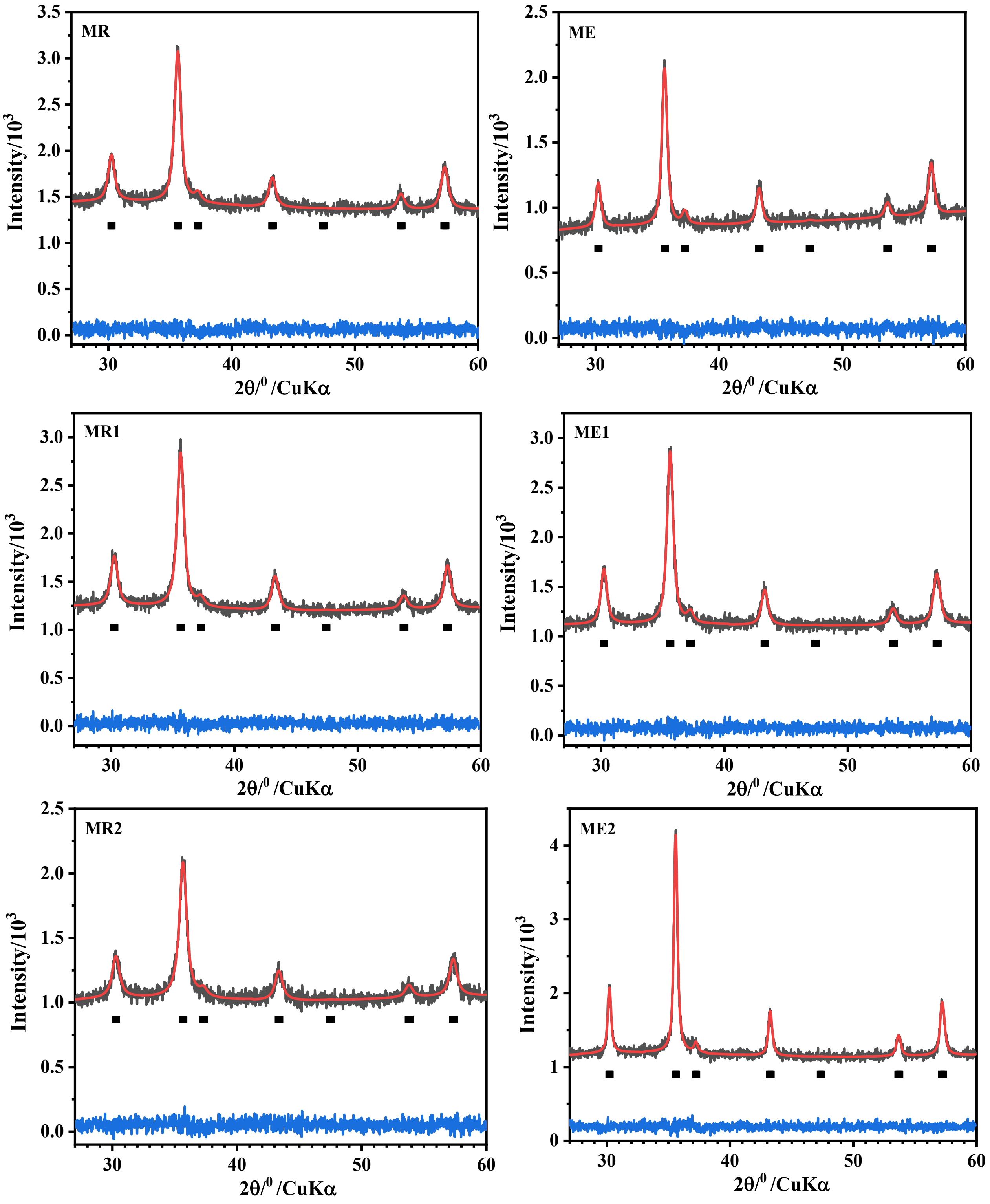

Figure S3. Powder X-ray diffraction patterns (black) of MR, MR1, MR2, ME, ME1, and ME2 after contact with $KMnO_4$. The solid red lines represent the profiles fitted by Rietveld refinement.
